# A machine learning and clustering-based approach for county-level COVID-19 analysis

**DOI:** 10.1371/journal.pone.0267558

**Published:** 2022-04-27

**Authors:** Charles Nicholson, Lex Beattie, Matthew Beattie, Talayeh Razzaghi, Sixia Chen

**Affiliations:** 1 School of Industrial and Systems Engineering, University of Oklahoma, Norman, Oklahoma, United States of America; 2 Data Science and Analytics Institute, University of Oklahoma, Norman, Oklahoma, United States of America; 3 Department of Biostatistics and Epidemiology, University of Oklahoma Health Sciences Center, Oklahoma City, Oklahoma, United States of America; National University of Sciences and Technology (NUST), PAKISTAN

## Abstract

COVID-19 is a global pandemic threatening the lives and livelihood of millions of people across the world. Due to its novelty and quick spread, scientists have had difficulty in creating accurate forecasts for this disease. In part, this is due to variation in human behavior and environmental factors that impact disease propagation. This is especially true for regionally specific predictive models due to either limited case histories or other unique factors characterizing the region. This paper employs both supervised and unsupervised methods to identify the critical county-level demographic, mobility, weather, medical capacity, and health related county-level factors for studying COVID-19 propagation prior to the widespread availability of a vaccine. We use this feature subspace to aggregate counties into meaningful clusters to support more refined disease analysis efforts.

## Introduction

The emergence of COVID-19 has evolved into a widespread pandemic in a very short time and drastically affected the United States and the world. Many forecasts are being made regarding the potential number of cases and fatalities associated with the virus. Much of the available data skew towards large urban areas. According to data available from John Hopkins University [1], as of September 6, 2020, there were 6,163,496 cases and 186,125 deaths in the US. Of those, 2,481,887 cases (40%) and 72,202 deaths (39%) were from the four most populous states (California, Texas, Florida, and New York). In contrast, a smaller state like Oklahoma had only 63,556 cases and 853 deaths. At the county level, the imbalance is even more explicit: 12 counties, less than 0.5% of all counties in the US, represent over 20% of total COVID-19 cases, and only 8 counties account for 20% of the reported deaths.

All projections of the spread of COVID-19 are subject to the limitations of the data upon which they are based. At the national level, projections are dominated by the volume of cases from large regions (states or counties). Projections for less populous areas become more difficult due to limited case histories and each location’s heterogeneity. These less populous areas also tend to be the least prepared for an onslaught of COVID-19 cases [2–4]. Hospitals and medical funding in these counties rely on forecasting to determine how to concentrate their efforts to prepare for a potential outbreak without depleting precious resources that can be used for other needs such as education. Given the skew of data towards urban areas, many forecasts for rural, semi-rural, and small populations result in over or under forecasting outbreaks. With limited economic resources, relying on inaccurate forecasting can result in unnecessary spending or, in the case of under forecasting, the loss of human lives.

Many traditional tools for disease analyses leverage only limited data to distinguish one area from another, i.e., age distribution and the number of current COVID-19 cases. While this may be sufficient to forecast disease spread for large regions, it is insufficient at a more refined level [5, 6]. For example, on April 10, 2020 using a Susceptible- Exposed-Infectious-Recovered (SEIR) model, the Oklahoma State Department of Health forecast that daily COVID-19 infections would peak in the state on April 21, and, by May 1, Oklahoma would have 9,300 total cases and 469 deaths [7]. In actuality, there were only 3,748 cases and 230 deaths by May 1, and the disease was nowhere near peaking.

Forecasting is complicated by the fact that critical variables can differ significantly geographically and demographically. That is, disease transmissibility is not only a characteristic of the biological pathogen, but also a function of human behavior and environmental factors [8, 9]. By not accounting for these differences, there is a risk of biasing the predictions towards large, urban areas and missing important unique traits among subgroups. The effect of this variation diminishes when considering large populations. However, there is a need for region-specific analyses and projections.

Additionally, while sufficient data quantity and quality might be available at higher levels of aggregation (e.g., state or country) or populous regions (e.g., New York City), this is not as likely at smaller scales and local levels. This study offers an approach to cluster small geographies based upon features found to be relevant to COVID-19 propagation. These clusters have greater amounts of data available for further modeling. To accomplish this, a large array of county-level data is collected for the 48 conterminous United States (US). Multiple machine learning approaches are used to analyze the data to discover the important and inherent county-level characteristics that potentially drive COVID-19 outcomes. The critical features are used to create clusters of counties with similar inherent traits. These clusters and their characteristics are anaylzed in detail. Ultimately, we propose that this approach provides a valid and beneficial compromise between the highly aggregated national or state level data and the more granular and limited local-level data.

## Related work

Multiple researchers and institutions have developed models for the spread of COVID-19, including publicly available tools from Stanford [10] and the US Center for Disease Control and Prevention [11]. A wide variety of propagation and forecasting models are being created alongside these since accurate prediction is proving to be a daunting task. The prediction models for the transmission dynamics of the COVID-19 pandemic can be categorized into two distinct classes: epidemiological methods and data-driven methods.

### Epidemiological models

The most common epidemiological models are compartmental models, which were first described in a series of three papers by Kermak and McKendrick in the 1920s and 1930s [12–14]. In these models, individuals in a population exist in and move between compartments: infected (I), susceptible (S), and recovered (R) individuals. The Susceptible-Infected-Recovered (SIR) [12] and Susceptible-Exposed-Infected-Recovered (SEIR) [15] models are among the most popular techniques for outbreak prediction since the onset of the pandemic [16–18]. Researchers continue to investigate enhancements for SIR and SEIR-based models. Sun et al. [19] proposed a novel SIR model with varying coefficients to track the reproductivity of the COVID-19 epidemic in China. Syage [20] considered a statistical and dynamical model for forecasting COVID-19 deaths based on a hybrid asymmetric gaussian and SEIR construct.

Compartmental models are useful for modeling the mechanisms of disease transfer, but they require the assumption of full-mixing within compartments and ignore many other factors such as geography, population heterogeneity, individual contact vectors, social dynamics, governmental decisions (e.g., lockdown measures), and other complexities of human behavior.

### Data-driven models

Data-driven models can provide more accurate forecasts at the expense of explicit modeling of propagation mechanisms. Methods such as agent-based simulation (ABS) [21] and machine learning (ML) methods have been employed for infectious disease outbreak analysis and disease prediction.

Agent-based simulation is a computer simulation approach consisting of agents (e.g., individuals) interacting with each other in a virtual environment. The advantage of ABS is that it can take into account a wide array of human-level dynamics while tracking disease spread. ABS has been applied for COVID-19 transmission modeling and prediction recently in [22–26]. While a powerful and flexible modeling paradigm, drawbacks of ABS include potential computational complexity, intricate modeling design assumptions, and the lack of closed-form “insight” on the observed system behavior.

The use of ML methods for COVID-19 forecasting is in its infancy. Yang et al. [27] developed the Long Short-Term Memory Networks (LSTM) to predict the COVID-19 epidemic using the 2003 SARS data as a training set. The COVID-19 epidemiological parameters, such as the probability of transmission, incubation rate, the probability of recovery or death and contact number, were used in the model. [28] proposed the use of 7 ML models and a new hybrid forecasting method based on nearest neighbors and *k*-means clustering to forecast COVID-19 growth rates. They employed LSTM, multiple linear regression, ridge regression, decision trees, random forest, neural network, and support vector machines on country level data (from the USA, India, UK, Germany, and Singapore). Other existing works have used the combination of epidemiological and machine learning models to predict pandemic propagation. [29] employed the SEIR model to obtain the value of *R*_0_ and then they predicted the number of COVID-19 confirmed cases in India for the next 21 days using regression.

County-level COVID-19 propagation modeling has proven to be challenging for multiple reasons. Disease transmission is influenced by “numerous biological, sociobehavioral, and environmental factors that govern pathogen transmission.” [8]. For instance [30], found that rural populations in China had a less positive attitude towards COVID-19 preventive behaviors and were less likely to adhere to policies such as social distancing and using masks. Some very recent work has begun to recognize the urgency of creating refined propagation models. Wang et al. [31] and Zhou et al. [5] are two examples that both address county-level spatiotemporal modeling to predict COVID-19 related outcomes.

### Research contribution

**Fig 1:**
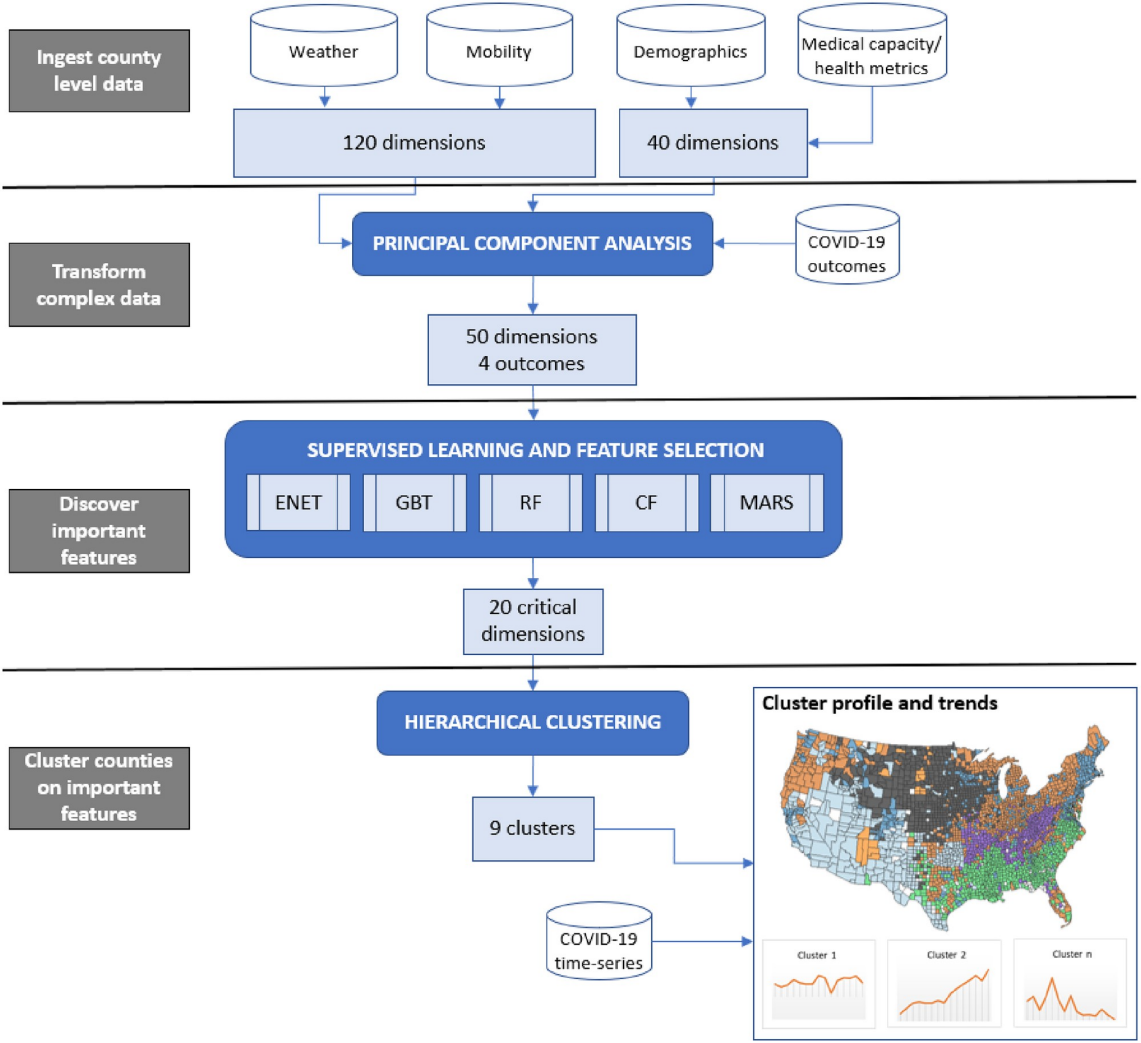
Machine learning to enhance county-level COVID-19 analyses.

This study contributes to the growing body of knowledge and methods for county-level infectious disease analysis in multiple ways. The primary objective is to discover the most important county-level characteristics relating to COVID-19 propagation and aggregate individual counties into clusters based on the important county-level characteristics. Ideally, this will help balance the issues associated with high-level aggregation (which hide regional diversity but have sufficient data for evaluating trends and creating forecasts) with the granular data at the local level (which has significant diversity but may have limited populations, cases, etc. for in-depth analysis). To achieve the overall objective, we complete four important subtasks, detailed below and depicted in Fig 1, that each contributes to literature.

First, we produce a unified county-level database for the US that includes demographics, mobility, weather, medical capacity, and health related county-level data relating to COVID-19 propagation. The data is available at http://oklahomaanalytics.com/software-research-data. Second, we extract essential information from the high dimensional weather and mobility data by projecting these features to a lower dimensional space to support meaningful clustering. Third, the resulting feature set is analyzed via supervised learning to discover the most important county-level characteristics relating to COVID-19 propagation. It is important to note that we are not performing time series forecasting or month-to-month predictions, but rather identifying the *underlying* traits, i.e., the aforementioned “sociobehavioral” and “environmental factors”, affecting COVID-19 outcomes. To the best of our knowledge, this level of in-depth and advanced empirical analysis of the critical county-level factors for COVID-19 is a novel contribution. Finally, balancing statistical properties and practical considerations, we aggregate individual counties into clusters based on the important county-level characteristics. This increases the amount of data available for epidemiological models yet the aggregation retains regional-level diversity on the critical features. Each cluster is profiled and analyzed to demonstrate the validity of the approach and to set the stage for future work. We believe that our analytical approach, list of important variables related to COVID-19 outcomes, and novel clustering results will provide important practical guidance for health policy makers and stakeholders to implement future intervention and resource allocation plans for COVID-19 and other infectious diseases.

## Data and methods

### Data

The data for this study is collected from multiple sources and includes demographic, health, mobility, and weather features for counties and county-equivalents across the US. The demographic data is gathered from a public data repository created by a group of faculty and students at John Hopkins University [32] that extracts and cleans data from various sources including the United States Census Bureau. The data reflects demographics as of 2017 or 2018 depending on the feature [33]. The relevant census data features include population, population by race and sex, population changes due to migration, number of births, number of deaths, and other descriptive demographic statistics. Population by race/ethnic data is aggregated to reflect the following categories: Hispanic alone, or non-Hispanic White, Black, Asian, Native Hawaiian or Pacific Islander, or Native American alone. Additionally, the multiple census categories regarding two or more races (whether Hispanic or not) is aggregated into a single category.

The health care variables concerning the number of beds, hospitals, admissions, and full-time employees are collected from the COVID Severity Forecast data set, which pulls said features from Kaiser Health News, Amma Resonance Healing Foundation Health [34], and the Behavioral Risk Factor Surveillance System. The mobility features are gathered from Google Mobility [35] and reflect monthly averages of daily metrics that describe how mobility changes against the counties’ baseline scores. Monthly averages are defined to help account for missing daily data for smaller counties. Weather features are sourced from the National Oceanic and Atmospheric Administration and accessed via the Google BigQuery Platform [36]. These features reflect monthly averages of high temperature, low temperature, average temperature, high humidity percentage, low humidity percentage, and average humidity percentage. Lastly, the COVID-19 case data is collected from USA Facts [37] and includes the number of confirmed cases and number of deaths by county starting in January 2020. This information is updated daily and this study uses data through October 10, 2020.

The data was merged from the various source based on the unique Federal Information Processing Standard code that uniquely identifies counties and county equivalents. All features are continuous numeric features. The values are standardized to represent Demographic, health-related, and COVID-19 case data are expressed per 1000 capita or as rates within the county population. The data set consists of 3,106 counties or county-equivalents (e.g., parishes and independent cities) across the conterminous US (two counties had missing data and the District of Columbia was not included). Each county is represented by 160 numerical features.

### Principal component analysis

Principal component analysis is a statistical technique used to project high dimensional data to lower dimensions in a way that preserves the original variance in the data [38]. The approach is commonly used in many fields to simplify data for human consumption or visualization, reduce inherent correlation in data sets, or to mitigate the so-called ‘curse of dimensionality’ associated with machine learning [39].

### Supervised learning and variable importance

Supervised learning is a class of machine learning algorithms that use a set of data points and known outcomes to determine a predictive model to map input space to outcomes. Many of these algorithms allow for complex, non-linear relationships between the input and outcome variables. While the resulting models may be difficult to interpret, the most important variables for predictive modeling can be identified, e.g., [40, 41]. The techniques selected each have rigorous, algorithm-specific mechanisms for quantifying the most important predictors. For instance, while support vector machines and neural networks are known to produce highly accurate models, neither have high quality methods to evaluate which predictors are the most important. Random forests, on the other hand, quantify individual variable importance naturally throughout the model building process. The methods, their hyperparameters, and the associated measure for determining variable importance are briefly described.

#### Elastic net regression

Elastic Net Regression (ENET) [42] is a penalized linear regression method that combines the *l*_1_-norm and *l*_2_-norm regularization elements of the least absolute shrinkage and selection operator method and ridge regression, respectively, to perform automatic feature selection and to reduce overfitting. The hyperparameters to be tuned include the penalty weight and the mixing parameter associated with balancing the *l*_1_ and *l*_2_ elements in the cost function (λ_1_ and λ_2_) in Eq (1). The absolute values of the *t*-values associated with the coefficients ${\hat{\beta }}_{ENET}$ are used to rank the variables in terms of importance.

$$\begin{array}{c} {\hat{\beta }}_{ENET}=(1+\frac{\lambda_{2}}{n})\left\{\underset{\beta }{\text{argmin}}{∥y-\sum_{j=1}^{m}x_{j}\beta_{j}∥}^{2}+\lambda_{1}{∥\beta ∥}_{1}+\lambda_{2}{∥\beta ∥}_{2}^{2}\right\} \end{array}$$ (1)

where *x*_1_, …, *x*_*m*_ are *m* predictors and *y* = (*y*_1_, …, *y*_*n*_)^*T*^ is the response variable for *n* observations.

#### Multivariate adaptive regression splines

Multivariate Adaptive Regression Splines (MARS), proposed by Friedman (1991) [43], construct a piecewise linear regression model by creating new features that isolate ranges of values from the original input data through the use of so-called hinge functions. Variables, their hinged-versions, and interactions between variables are sequentially added to a linear regression model. Once complete, MARS employs a backwards stepwise elimination procedure to reduce the number of features and optimize the generalized cross-validation (GCV) performance statistic. The hyperparameters relate to the allowed degree of variable interaction and the maximum size of predictors allowable after this second step. Variable importance is determined during the backwards elimination procedure and based on the effect that the presence of a given variable has on the GCV value.

The MARS-based model can be formulated as shown in Eq (2):

$$\begin{array}{c} y=\delta_{0}+\sum_{p=1}^{P}\delta_{p}h_{p}\left(X\right) \end{array}$$ (2)

where *h*_*p*_(*X*) are spline functions, *P* is the number of spline functions, X is the predictor set, y is the response variable, *δ*_0_ represents the constant coefficient, and *δ* are coefficients that are computed from the sum of squared errors minimization problem. MARS is a popular variable selection method since it does not consider any assumption about the data distributions and nonlinear associations between the variables [44], which makes it effective in modeling complex nonlinear relationships such as COVID-19 occurrence and death.

#### Random forests, conditional inference forests, and gradient boosted trees

Random forests (RF) [45], conditional inference forests (CF) [46], and gradient boosted trees (GBT) [47] each leverage an ensemble of weak learners (i.e., decision trees) to create highly predictive regression and classification models. RF and CF create many independently constructed decision trees and use a majority rule to determine outcome values. To reduce inter-tree correlation, at each step during the tree building process, only a random subset of predictors are evaluated to create node splits. RF uses an impurity metric to determine the split values whereas CF employs statistical tests. The number of variables considered at each split is tuned to reduce overfitting.

GBT constructs a sequence of simple decision trees in which each tree is built based on the results of the previous tree predictive error. Hyperparameter values include the number of trees to fit, the maximum depth of each tree, the learning rate, and the minimum number of observations in the terminal nodes of the trees. For both RF and CF, the mean-squared error (MSE) on the out-of-bag data is recorded for each tree and each variable. Variables that most improve the MSE have higher importance scores assigned. For GBT, variable importance is related to how often a feature is selected in the construction of underlying trees.

### Clustering

Clustering is an unsupervised machine learning approach to identify clusters of observations within data such that the intra-cluster similarity is high and the inter-cluster similarity is low. Suppose that a data set is represented by a set $D={\left\{\left(x_{i}\right)\right\}}_{i=1}^{n}$ where $x_{i}\in R^{m}$, such that there are *n* observations and each *x*_*i*_ is a observation with *m* features. Assume a set of *k* clusters **C** = {*C*_*j*_;*j* = 1, …, *k*} in which *k* is a predefined parameter. In this study, we use three clustering algorithms, namely: *k*-means [48, 49], partitioning around medoids (PAM) [50, 51], and hierarchical clustering (HC) [52, 53]. These three methods rely on distance measures between objects in a data set. We use Euclidean distance on mean-centered and scaled variables (scaled with respect to each feature’s observed standard deviation). All three methods require user input with respect to the number clusters to be identified.

#### *k*-means

*k*-means is a popular clustering algorithm proposed by [54]. The goal of *k*-means is to obtain a partition that minimizes the squared error between the mean of a cluster and the observations within that cluster. For a cluster *C*_*j*_, the squared error between the mean of the cluster, $\mu_{C_{j}}$, and all the observations in the cluster is given by Eq (3):

$$\begin{array}{c} J\left(C_{j}\right)=\sum_{x_{i}\in C_{j}}{∥x_{i}-\mu_{C_{j}}∥}^{2}. \end{array}$$ (3)

Then, the partition is identified from the solution of the following unconstrained minimization problem over all *k* clusters:

$$\begin{array}{c} J\left(C\right)=\sum_{j=1}^{k}\sum_{x_{i}\in C_{j}}{∥x_{i}-\mu_{C_{j}}∥}^{2}. \end{array}$$ (4)

#### Partitioning around medoids

The partitioning around medoids (PAM) algorithm is the most widely known implementation of *k*-medoid clustering [55]. The advantage of the PAM method compared to other clustering methods is its robustness towards outliers [56] and flexibility to allow the use of various types of variables such as categorical and numeric variables [57]. It aims to find a good partition using *k* representative observations *m*_*j*_ (*j* = 1, 2, …, *k*) called medoids. The medoid of a set *C*_*j*_ is defined as the observation with the smallest sum of dissimilarities/distances to all other observations in the set according to (5):

$$\begin{array}{c} m_{j}=\underset{x_{i}\in C_{j}}{\text{argmin}}\sum_{x_{j}\in C_{j}}{∥x_{i}-x_{j}∥}^{2}. \end{array}$$ (5)

Then, the *k*-medoid generates *k* clusters in an iterative algorithm such that the total distances from each observation to its cluster’s medoid over all *k* clusters is minimized as follows:

$$\begin{array}{c} J\left(C\right)=\sum_{j=1}^{k}\sum_{x_{i}\in C_{j}}{∥x_{i}-m_{j}∥}^{2} \end{array}$$ (6)

PAM selects the medoids for each cluster using two phases called *build* and *swap*. The build phase finds an initial clustering through the consecutive selection of *k* medoids. The swap phase improves the selected set of medoids and then finds the clustering in an iterative process until the objective function value shown in Eq (6) no longer decreases or there is no further update in the set of medoids between two subsequent iterations.

#### Hierarchical clustering

Hierarchical clustering techniques iteratively find nested clusters by constructing a tree structure either in agglomerative (bottom up) or divisive (top down) manner. Agglomerative clustering begins with each observation in its own cluster and subsequently combines the least dissimilar pair of clusters into a single cluster, thus producing a hierarchy. In this study, we use agglomerative clustering because it is the most popular and practical approach. There are different measures to obtain the distance between clusters such as single linkage, complete linkage, and Ward’s method [58]. We choose the latter for this study as it is based on minimizing the within sum of squares error from Eq (3) at iteration when combining clusters.

Let *C*_*i*_ and *C*_*j*_ denote two mutually exclusive clusters consisting of *n*_*i*_ and *n*_*j*_ points, respectively. Let *d*(*C*_*i*_, *C*_*j*_) denote the dissimilarity between *C*_*i*_ and *C*_*j*_. Ward’s method computes dissimilarity as the increase in the sum of squares if *C*_*i*_ and *C*_*j*_ are merged. Mathematically, this is equivalent to

$$\begin{array}{c} d_{\text{Ward}}\left(C_{i},C_{j}\right)=\frac{n_{i}n_{j}}{n_{i}+n_{j}}{∥\mu_{C_{i}}-\mu_{C_{j}}∥}^{2}, \end{array}$$ (7)

where $\mu_{C_{i}}$ and $\mu_{C_{j}}$ are the means of clusters *C*_*i*_ and *C*_*j*_, respectively.

### Computational tools

All statistical analysis, supervised learning, and clustering is performed using the R software environment [59] and the following R packages: elastic net models are developed using *glmnet* [60], the random forests are developed using *randomForest* [61], the conditional inference forests are developed using *partykit* [62], the gradient boosted trees are developed using *gbm* [63], and the MARS models are developed using *earth* [64]. Cross-validation is conducted using the *caret* package [65]. Finally, the mapping is performed using the package *usmap* [66].

## Results

### Dimension reduction

For each county and each month, the average, minimum, and maximum temperatures and relative humidities are reported, producing 72 dimensions of data. For the mobility data, the changes are reported with respect to grocery, park, retail, residential, transits, and workplace values for February 2020 through September 2020, generating 48 dimensions. The weather variables exhibit high correlation with each other, as do the mobility variables. Both the weather and mobility data can be projected onto considerably lower dimensions while maintaining the majority of their informational value. Indeed, this finding is important for the success of the research effort. Ideally, we desire all of the input variables for the clustering procedure to represent inherent traits associated with each county. For example, we prefer general county-level weather characteristics (e.g., colder than the average US county) over a historical month’s specific values (e.g., the high temperature in May 2020). The former is easy to generalize, but the latter is not. We would like to project mobility data in a similar way—i.e., compacting the month-to-month specific data into something that relates to an overall behavioral pattern. Fortunately, the high correlation of variables indicates that this is feasible with principal component analysis.

**Fig 2:**
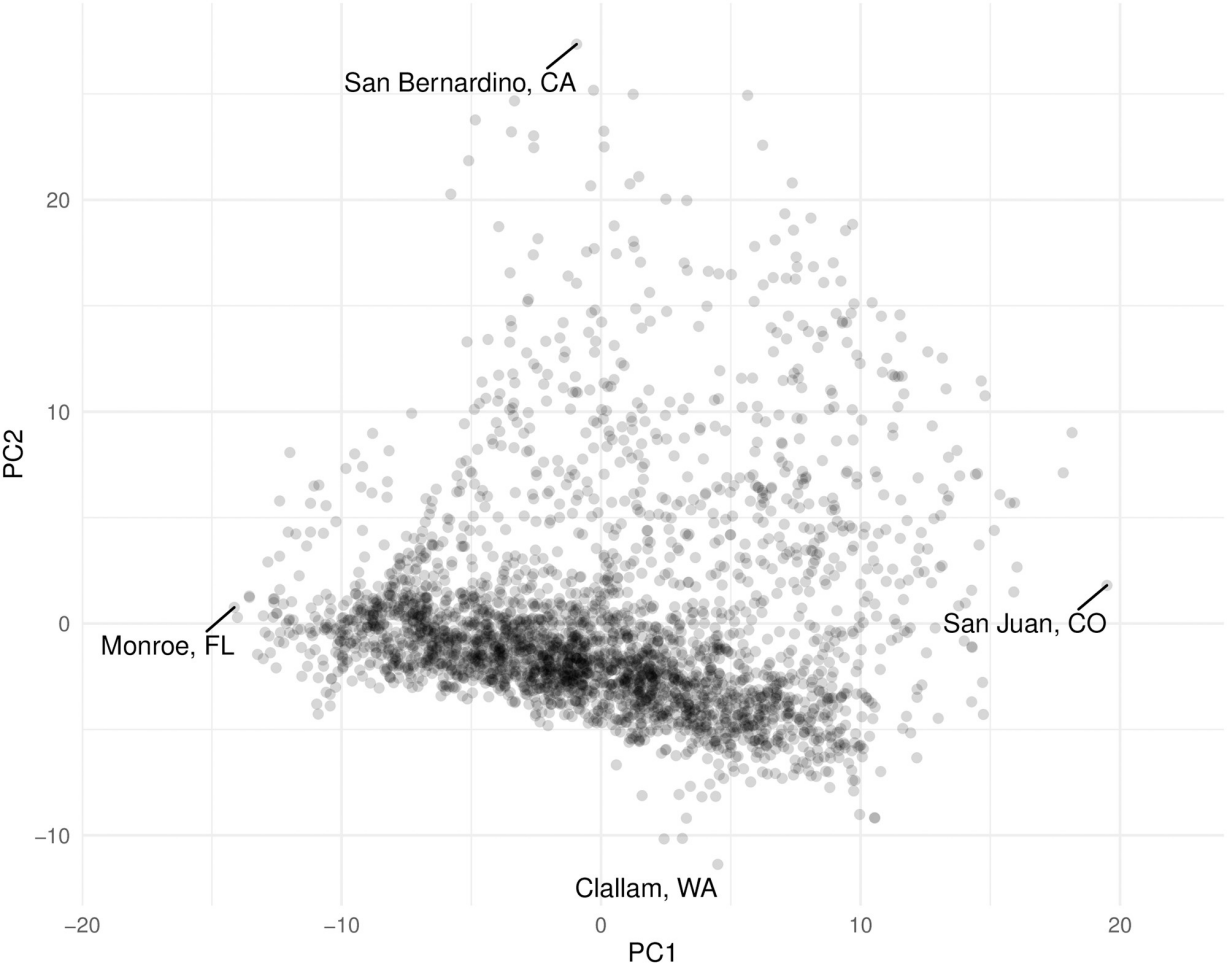
County-level weather data projected to 2 dimensions with PCA.

Using PCA, the weather data is first mean-centered and scaled with respect to feature standard deviation. Next, the data is projected from 72 dimensions to 2 principal components while retaining approximately 80% of the original variation. The first principal component (PC1) explains 47% of the variance and is dominated by the monthly temperature related variables. The second principal component (PC2) explains 33% of the variance and is dominated by the monthly humidity related variables. The 2D projection is depicted in Fig 2. The counties associated with extreme values for each axis are labeled. The mean-centered and scaled 48 dimensional mobility change data is successfully projected onto 8 dimensions while retaining nearly 80% of the original variance.

### COVID-19 supervised learning and variable importance

Each of the supervised learning approaches described beforehand is trained to predict four distinct county-level outcomes: total per 1000 capita positive COVID-19 cases as of October 10, 2020 (*cases*), total per 1000 capita COVID-19 deaths as of October 10, 2020 (*deaths*), the growth rate for positive cases over the most recent 30 days (September 11, 2020 to October 10, 2020) (*case rate*), and the growth rate for COVID-19 deaths over the same 30 days (*death rate*). The goal of the training is to identify which county-level variables are the most important driving factors associated with COVID-19 outcomes. Table 1 summarizes the four target variables.

**Table 1:** COVID-19 outcomes per county.

Target variable	Description
*cases*	total positive COVID-19 cases per 1,000 capita
*deaths*	total COVID-19 deaths per 1,000 capita
*case rate*	30-day average of new COVID-19 cases per day per 1,000 capita
*death rate*	30-day average of new COVID-19 deaths per day per 1,000 capita

The models are trained on the county-level aggregated data set and tuned using 5-fold cross-validation with five repeats. The minimal cross-validated (CV) root mean squared error (RMSE) is used to determine the associated hyperparameter values and to evaluate the generalizable error of each model. Table 2 reports the predictive performance for each model. For each outcome variable and supervised learning method, the average CV RMSE and average CV *R*^2^ metrics are listed. The RMSE values provide an effective method for comparing models for a given outcome and are listed first; the *R*^2^ values facilitate comparisons between models of different outcomes and are listed below the RMSE values. For each outcome predicted, the performance values associated with the model having the lowest CV RMSE values are in bold.

**Table 2:** Model performance.

Outcome	Metric	Supervised learning method				
		ENET	RF	CF	GBT	MARS
*cases*	RMSE	11.2616	**9.9849**	10.2133	10.0319	10.9266
	*R* ^2^	0.4428	**0.5704**	0.5510	0.5584	0.4808
*deaths*	RMSE	0.4279	**0.4160**	0.4164	0.4173	0.4338
	*R* ^2^	0.4070	**0.4586**	0.4389	0.4356	0.3897
*case rate*	RMSE	0.1482	**0.1421**	0.1436	0.1425	0.1505
	*R* ^2^	0.2852	**0.3521**	0.3423	0.3395	0.2659
*death rate*	RMSE	0.0054	0.0054	**0.0053**	0.0054	0.0055
	*R* ^2^	0.1096	0.1060	**0.1259**	0.1193	0.0919

ENET and MARS generally underperform on all outcomes with respect to the RF, CF, and GBT algorithms. This implies that the fundamental relationships between the county characteristics and COVID-19 outcomes are both complex and non-linear. For predicting *cases*, *deaths*, and *case rate*, the random forest model performs the best. The conditional inference forest outperforms the competing techniques when predicting *death rate*. Each of the four forest methods are built with 500 trees. The tuned hyperparameter values for the four best models define the number of variables considered at each split of the underlying trees. For all four models, this value is tuned using cross-validation and found to range from 10-20.

In terms of overall predictability, the highest CV *R*^2^ is 0.5704 using a random forest model to predict *cases*. It is important to note that this model uses only non-pathogen characteristics and no historical case load information, yet it captures over 57% of the variation in COVID-19 cases. The best predictive performances correspond to predicting the per capita cases by county. The next best set of predictive models are associated with *deaths*. The models predicting *case rate* are next with *R*^2^ values in the range of 0.2659 to 0.3521. Finally, every technique applied has difficulty predicting the increase in COVID-19 deaths for the most recent 30 days. This may be due to an inherent lack of predictability (e.g., due to noise in the data) or indicative that there are important features missing from the collected data.

To identify the critical county-level factors, the top 10 variables, ranked in terms of variable importance, for each of the best predictive models in Table 2 are extracted. Since multiple variables are important in different models, this set is comprised of 20 distinct variables. These 20 critical features are listed, categorized, and described in Table 3. Four race/ethnicity variables are important: non-Hispanic Whites, Blacks, and American Indian (alone) and the per capita number of individuals belonging to two or more races (regardless of Hispanic classification). In terms of medical capacity, the number of specialized nursing facilities (including nursing homes) and the ratio of insured to uninsured individuals is critical. Three health related factors are identified as critical: percent of individuals who self-report as being in fair or poor health, the number of self-reported mentally unhealthy days, and the percent of the county that are smokers. The county-level median income and unemployment rate are two important economic factors. The first two principal components derived from the weather data are top predictors. Education level, age brackets, and population density each make the list as well as the ratio of Democrats to Republicans in each county.

**Table 3:** Important variables for county-level COVID-19 modeling.

Variable category	Name	Description
Race/ethnicity	NHWA	not Hispanic, White alone
	NHBA	not Hispanic, Black alone
	NHIA	not Hispanic, American Indian alone
	TOM	two or more races
Medical capacity	SNF-sites	specialized nursing facilities per capita
	health-insurance	ratio of insured to uninsured (for ages 40-64)
Health	pct-FairPoorHealth	percent reporting fair or poor health
	days-UnhealthyMental	self-reported mentally unhealthy days
	pct-Smokers	percent who smoke
Economics	median-income	median household income
	unemployment-rate	percent of labor force that is unemployed
Weather	PC1-wx	PC1 for weather data
	PC2-wx	PC2 for weather data
Education	pct-woHSdiploma	percent adults without HS diploma
	pct-4yr-degree+	percent adults with 4 yr degree or higher
Age	under18	population under 18 years of age
	over65	population over 65 years of age
Gender	gender-ratio	ratio of males to females
Density	pop-density	population density (per square mile)
Politics	dem-rep-ratio	ratio of Democrats to Republicans

**Fig 3:**
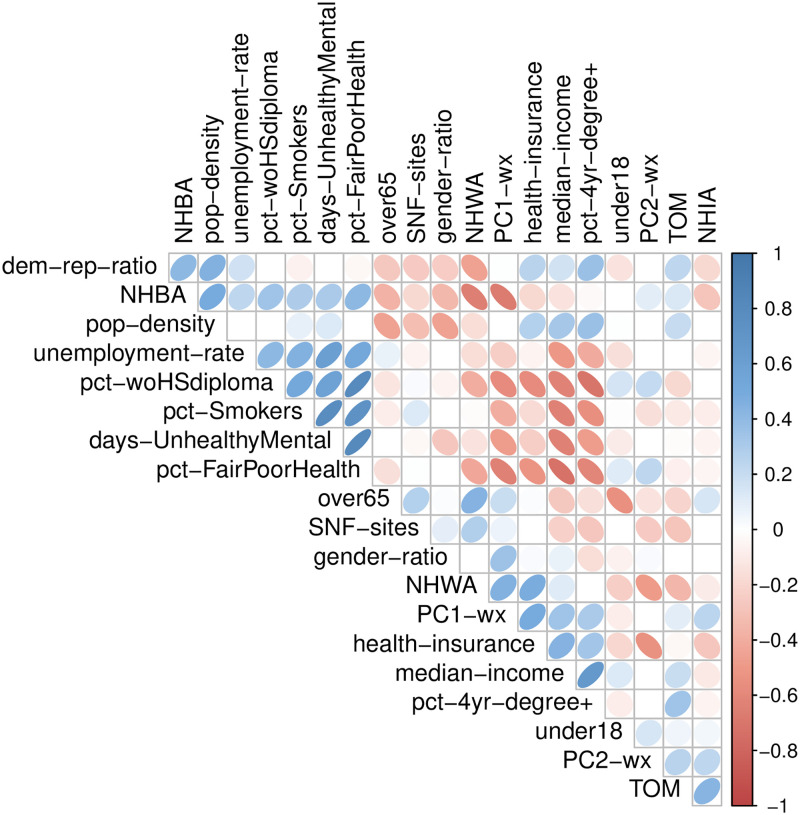
Correlation plot for critical COVID-19 county-level variables.

Fig 3 depicts a Spearman’s *ρ* rank correlation plot for the 20 variables reported in Table 3. The correlation strengths are represented by ellipses in each cell. Strong correlations are indicated by dark, thin ellipse angled to the right (positive correlation) or to the left (negative correlation). Statistical tests for the correlation values are conducted at a significance level of 0.05. If a correlation is not statistically significant at this level, the corresponding cell is left blank.

Multiple variables demonstrate levels of moderate to strong correlation (or anti-correlation). The unemployment rate, percent of county without a high school degree, percent of county that are smokers, self-reported unhealthy mental days and self-reported fair/poor health status form a group of positively rank correlated variables. These same variables are negatively rank correlated to the set of factors including median income, percent of county with a 4-year degree and to some extent, with the ratio of health insurance, the first principal component for weather data, and the population of non-Hispanic Whites. The non-Hispanic Black population is negatively rank correlated with the first principal component for weather data and the population of non-Hispanic Whites, but positively rank correlated with the Democrat to Republican ratio and population density.

Table 4 lists the top ten variables, in order of importance, for each of the top performing models used in the prediction of the four distinct outcomes. The individual variable importance scores, scaled between 0 and 100 and rounded to the nearest integer, are reported in parentheses. The weather factor is a prominent predictor in all four models and the most important in all but the *case rate* model. This may reflect geographic diversity across the US and/or a more typical influenza-like propagation behavior associated how individuals spend more time indoors during inclement weather. Racial factors also play an important role in all four models. The *deaths* model uses all four race/ethnicity indicators. The *case rate* and *death rate* models only consider one race variable each, non-Hispanic American Indians and non-Hispanic Blacks, respectively. It is of note that self-reported mentally unhealthy days is the most important variable for the *case rate* model. This feature correlates (positively or negatively) with other socioeconomic factors such as percent reporting fair or poor health, median income values, health insurance coverage, and education. It may be that self-reported unhealthy days is an indication of other unhealthy behaviors or conditions that could lead to increases in COVID-19 cases. It is interesting that the *death rate* model has median income as its second most important variable and together with the *case rate* model are the only two models that identify the number of SNF sites and health insurance status as important predictors.

**Table 4:** Important variables by model.

cases	deaths	case rate	death rate
PC1-wx (100)	PC1-wx (100)	days-UnhealthyMental (100)	PC1-wx (100)
NHWA (65)	NHBA (93)	unemployment-rate (47)	median-income (84)
pct-woHSdiploma (46)	NHWA (89)	NHIA (46)	days-UnhealthyMental (70)
pct-FairPoorHealth (34)	TOM (37)	SNF-sites (42)	NHBA (52)
gender-ratio (31)	pct-woHSdiploma (35)	PC1-wx (36)	SNF-sites (45)
NHBA (31)	pct-FairPoorHealth (31)	pct-Smokers (35)	pct-FairPoorHealth (44)
days-UnhealthyMental (30)	NHIA (28)	under18 (21)	health-insurance (39)
under18 (30)	gender-ratio (26)	dem-rep-ratio (21)	pct-4yr-degree+ (33)
pct-Smokers (24)	median-income (22)	PC2-wx (20)	pct-Smokers (29)
over65 (24)	pop-density (18)	health-insurance (19)	pct-woHSdiploma (23)

### County-level clustering

The 20 features identified as critical intentionally do not include any direct COVID-19 outcomes. The objective is to identify county-level characteristics that are fundamental factors impacting how COVID-19 spreads within a community. If successful, identifying clusters of counties within this 20 dimensional subspace may enhance future analysis methods and allow researchers to distinguish important trends.

#### Number of clusters

To create the subgroups, *k*-means, PAM, and agglomerative hierarchical clustering (HC) results are extensively evaluated on the mean-centered and scaled data. Simulation studies have shown there is no best clustering algorithm that works for all scenarios [67–69]. The appropriateness of a particular algorithm is dependent on the nature of the data and on the information sought. For example, *k*-means and PAM tend to produce “spherically” shaped clusters, whereas hierarchical clustering does not have a similar limitation. When *a priori* knowledge about the data is not available or insufficient, it is common to explore different algorithms to obtain meaningful clustering results through comparisons. The final choice should be a balance between statistical properties and practical interpretation.

The choice of the number of clusters is also somewhat subjective. There are many quantitative index methods used in the literature to identify the appropriate number of clusters. Unfortunately, these indicators do not typically agree with one another and there is no single “correct” method for determining the right cluster quantity. This discrepancy is clear from the excerpt of indices shown in Table 5 for *k*-means, PAM, and HC with the county-level data. A missing value in the table denotes that the index does not apply or is not commonly used for the associated clustering method.

**Table 5:** Recommended number of clusters.

Index name	*k*-means	PAM	HC
Beale [70]	2	.	11
DB [71]	15	.	12
Silhouette [72]	2	2	2
Marriot [73]	7	.	6
Point-biserial [74]	4	.	7
Gap statistic [75]	6	2	6 to 11

**Fig 4:**
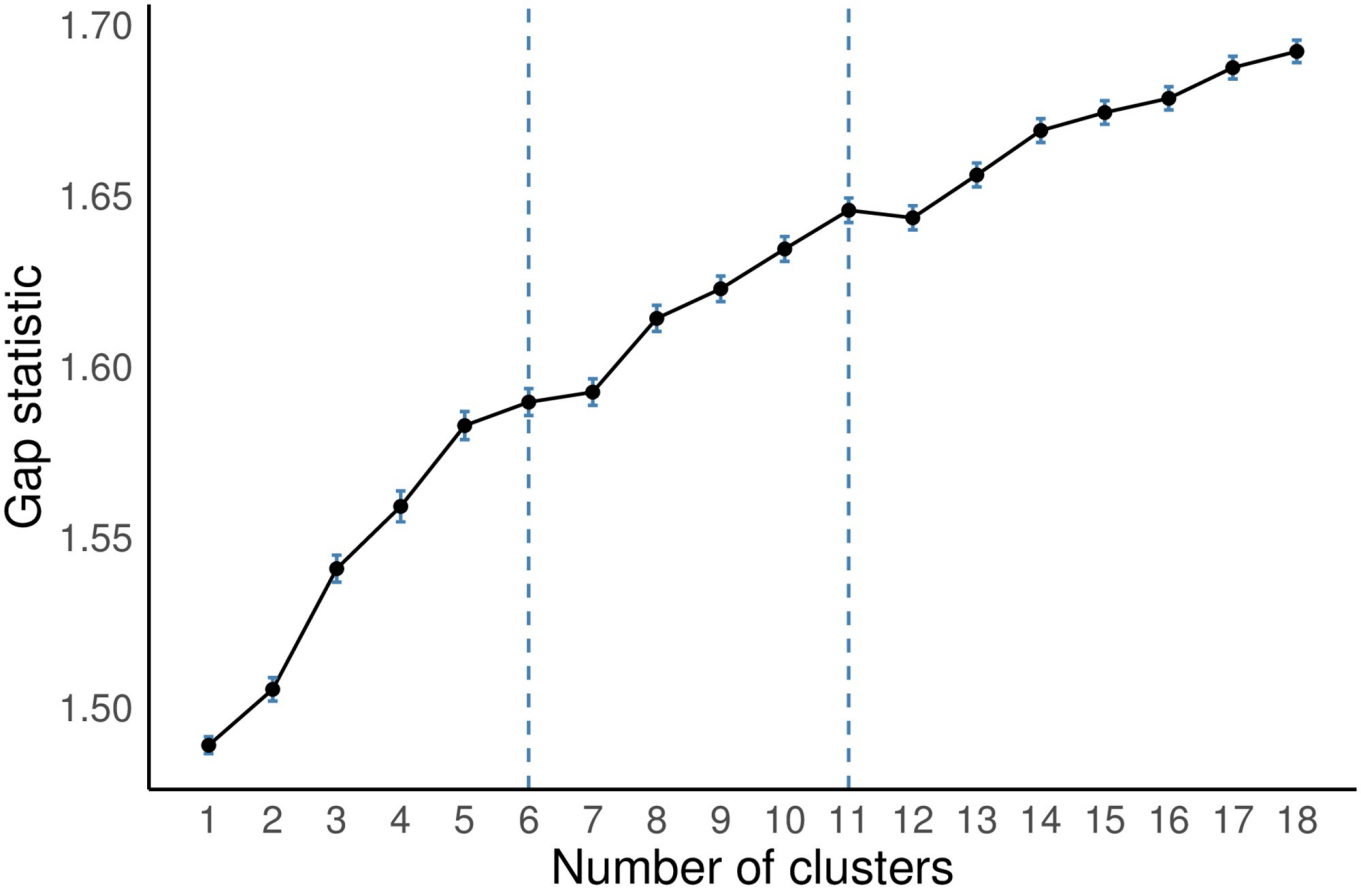
Gap statistic for the hierarchical clustering.

The Gap statistic is a modern numeric approach leveraging Monte Carlo simulation to help determine the optimal number of clusters and is applicable to *k*-means, PAM, and HC. Simulation studies shows that the gap statistic outperforms other early methods [75]. The results indicate that 2, 6, or values from 6 to 11, are good settings for *k*, respectively, for the three algorithms. Fig 4 depicts a plot of the gap statistic means and standard errors using 500 bootstrapped samples for *k* = 1, …, 18 for the hierarchical cluster values. The lower value of *k* = 6 is determined based on the guidance from [75], which considers the observed standard errors. The higher value of *k* = 11 is determined from the location of the first local maximum in the Gap statistic graph. Given the inconsistency from the index methods, we take the recommended value from the more modern Gap statistic to produce clusters for analysis. After visual inspection and evaluation of the characteristics of many sets of identified clusters, we choose the HC clusters with *k* = 9 as a good balance to support the objectives of this study, i.e., to identify clusters of reasonable size and similarity that also reflect a level of regionally specific diversity that can be leveraged to support public health decision-making.

#### Cluster geographic description

**Fig 5:**
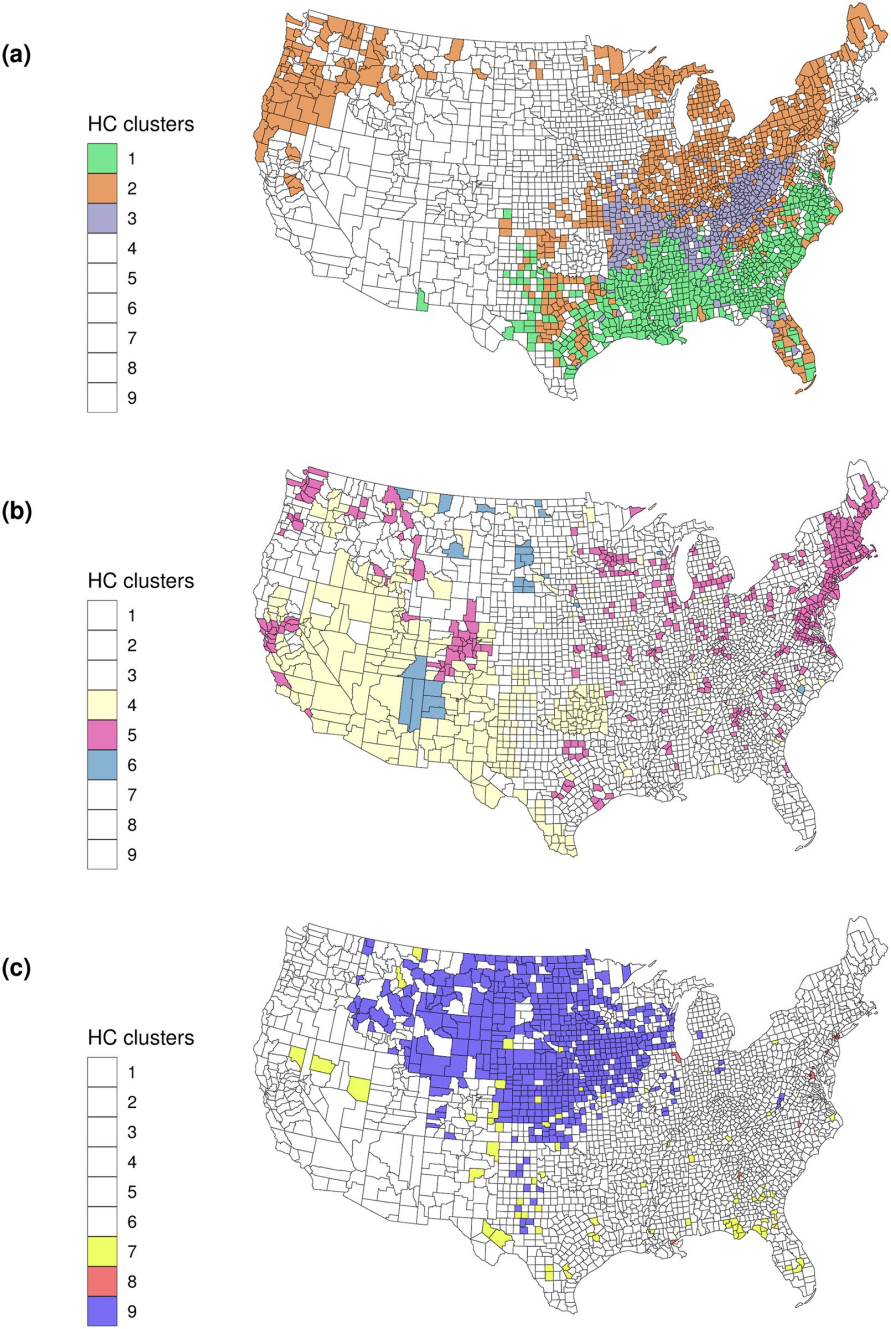
Conterminous US with county-level cluster assignments.

Fig 5 depicts the geographical locations of the nine clusters. For clarity, the figure is shown in three maps, the first depicts clusters 1, 2, 3; the second depicts clusters 4, 5 and 6; and the third depicts clusters 7, 8, and 9. While each cluster is often formed by sets of contiguous counties, this is entirely the result of inherent regional similarities along the 20-dimensional critical subspace.

Cluster 1 is primarily spread throughout the Southern US census region; cluster 2 is widely dispersed and includes counties from the northwestern US, central Texas, western Oklahoma, Florida, and parts of the Northeastern US; cluster 3 forms a relatively tight grouping of counties primarily dispersed across parts of Arkansas, Missouri, Tennessee, and Kentucky. Cluster 4 is focused in mostly in the south part of the Western US region, cluster 5 is located across the US but especially grouped in certain areas (e.g., around the San Francisco area, Denver, and in the Northeastern states), whereas cluster 6 which is composed of only 24 counties, is located in small pockets of large area counties. Cluster 7 is another small cluster of mostly individual counties across the nation. Cluster 8 pinpoints specific, high population density counties such as San Francisco County, CA, and Bronx, NY. Cluster 9 is primarily located in the Midwestern US census region.

#### Cluster profile

The nine clusters are fully profiled in Table 6. For each cluster, the number of associated counties is reported along the average of the mean-centered and scaled values for each of the 20 critical dimensions. Additionally, the table reports the cluster average for the scaled COVID-19 outcomes, i.e., cases, deaths, case growth rate, and death growth rate. The average scaled absolute values that exceed 1 are highlighted in bold. These values indicate that the average value within the associated cluster are greater than 1 standard deviation above/below the average for counties across the US. A brief description highlighting some discriminating attributes of each cluster follows.

- Cluster 1 has a larger Black population than the average and below average population for other races, especially White. This cluster also has a below average PC1-wx score indicating that it is associated with warmer regions. It has an above average score for the per capita COVID-19 cases and deaths and while its more recent case growth rate is about average, it has the highest value in the recent growth of COVID-19 deaths.
- Cluster 2 is the largest subset of counties from all the groups, and none of its scores are far from the overall national average.
- Cluster 3 has high scores for all three unhealthy metrics. This cluster has the highest score for the population of Whites and has one of the lowest median income values and a relatively low education level. This group has below average COVID-19 cases and deaths and is only slightly above average with respect to recent increases in either outcome.
- Cluster 4 has more population identifying with two or more races, is younger, and in colder region of the US than the average.
- Cluster 5 has the greatest median income and education levels and has among the lowest values for recent trends in COVID-19 cases or deaths.
- Cluster 6 has an American Indian population that is 9.5 standard deviations above the average for US counties. It also has the lowest median income, highest unemployment rate, lowest health insurance ratio, and some of the most unhealthy metrics for physcial and mental health. This group of counties has a population that is much younger than the average. The number of COVID-19 cases and recent COVID-19 case growth exceeds 1 standard deviation above the mean for all US counties. Cluster 6 has the highest values for the recent trend in COVID-19 deaths.
- Cluster 7 has the highest percentage of adults without a highschool degree and a much greater than average ratio of males to females (exceeding 4 standard deviations above the mean). This subset of 70 counties, has on average the highest per capita COVID-19 cases and above average values for the other three COVID-19 outcomes.
- Cluster 8 contains 21 counties whose average population density is far greater than the average (more than 7 standard deviations above the mean). It is cluster with the greatest Black population per capita, the highest ratio of Democrats to Republicans, and the highest college education level. While its per capita COVID-19 deaths to-date is the highest among all clusters, it has the lowest value for recent trend in COVID-19 cases and second to lowest in recent trend of COVID-19 deaths.
- Cluster 9 has the second highest score for White population and the lowest number of mentally unhealthy days and lowest value for self-reported Poor/Fair health. This group also reports the lowest unemployment rate from among all the clusters. It has the second highest recent COVID-19 case growth.

**Table 6:** Cluster profile.

Cluster	1	2	3	4	5	6	7	8	9
Number of counties	570	791	350	319	411	24	70	21	550
Scaled feature averages									
NHWA	**-1.03**	0.48	0.73	-0.97	0.08	**-2.48**	-0.67	**-2.19**	0.69
NHBA	**1.53**	-0.35	-0.40	-0.41	-0.16	-0.53	0.34	**2.03**	-0.56
NHIA	-0.18	-0.11	-0.19	0.52	-0.20	**9.50**	-0.09	-0.23	-0.08
TOM	-0.31	-0.02	-0.39	**1.12**	0.34	**1.02**	-0.22	0.63	-0.35
SNF-sites	-0.21	-0.07	-0.05	-0.35	-0.53	-0.53	0.18	-0.66	0.98
health-insurance	-0.70	0.21	-0.18	-0.69	0.99	**-1.10**	-0.64	0.10	0.33
pct-FairPoorHealth	0.90	-0.26	0.93	0.47	-0.89	**1.68**	0.59	0.20	-0.92
days-UnhealthyMental	0.56	0.14	**1.30**	0.10	-0.63	**1.13**	-0.04	-0.15	**-1.24**
pct-Smokers	0.49	-0.01	**1.24**	-0.32	-0.83	**3.17**	0.40	-0.20	-0.66
median-income	-0.60	-0.07	-0.81	-0.06	**1.60**	**-1.08**	-0.66	0.62	0.20
unemployment-rate	0.45	0.18	0.56	0.15	-0.56	**1.46**	-0.10	0.14	-0.79
PC1-wx	**-1.14**	0.13	-0.41	0.25	0.32	**1.24**	-0.68	-0.29	0.92
PC2-wx	0.02	-0.36	-0.37	**1.64**	-0.17	0.66	0.66	0.13	-0.20
pct-woHsdiploma	0.77	-0.27	0.74	0.68	-0.88	0.45	**1.05**	0.20	-0.77
pct-4yr-degree+	-0.42	-0.12	-0.76	-0.22	**1.67**	-0.57	-0.87	**1.78**	0.04
under18	0.13	-0.40	-0.27	**1.03**	-0.18	**3.00**	**-1.11**	-0.50	0.17
over65	-0.30	0.40	0.29	-0.53	-0.58	**-1.43**	-0.45	**-1.24**	0.46
gender-ratio	-0.27	-0.09	-0.19	0.17	-0.23	-0.16	**4.38**	-0.81	0.08
pop-density	-0.05	-0.06	-0.11	-0.10	0.20	-0.14	-0.14	**7.14**	-0.13
dem-rep-ratio	0.21	-0.19	-0.44	0.03	0.53	0.85	-0.29	**7.12**	-0.34
Scaled outcome averages									
*cases*	0.79	-0.46	-0.10	0.15	-0.38	**1.17**	**1.37**	0.36	-0.15
*deaths*	0.86	-0.24	-0.19	-0.06	-0.15	0.86	0.35	**1.56**	-0.41
*case rate*	-0.09	-0.25	0.11	0.02	-0.42	**1.56**	0.24	-0.54	0.61
*death rate*	0.41	-0.14	0.09	-0.06	-0.36	0.43	0.37	-0.32	-0.04

Clusters 6, 7, and 9 consist of counties with low population density, e.g., Big Horn, MT, Alfalfa, OK, and Kit Carson, CO, with 2.6, 6.5, and 3.8 persons per square mile, respectively. These rural clusters have greater than average recent COVID-19 case growth and/or recent increase in per capita deaths. Cluster 9 in particular is notable in that it represent 550 counties and while its per capita COVID-19 cases and deaths are lower than average, its recent above average increase in cases may precede a significant increase in COVID-19 deaths. Cluster 6 on the other hand, while rural and also colder than average, looks very different than cluster 9. Cluster 6 has a notable American Indian population and has the lowest median income, highest unemployment rate, lowest health insurance ratio, and some of the unhealthiest metrics in the data. Cluster 9 mostly represents White population with the least number of mentally unhealthy days and lowest values for self-reported poor/fair health. Our results with cluster 6 are consistent with previous studies that show COVID-19 incidence is much higher among American Indians/Alaska Natives than among White counterparts [76]. The lower values for the cluster 6 health and insurance factors imply that its recent case growth may have a more severe impact on lives lost. Indeed, the per county average for increase in recent deaths is already well above average.

The 7-day rolling averages of new COVID-19 cases per 100,000 capita for the combined populations of each cluster are depicted in Fig 6 from July 2020 until mid-October. The upticks in both cluster 6 and 9 are notable in that the other clusters have had relatively flat trends recently whereas these two have seen a pronounced increasing trend for several weeks. We hypothesize that the COVID-19 cases in both clusters have increased (since September) due in part to colder weather and potentially less restrictive lockdown policies. Cluster 6 has unique issues with inequities in access to health care, education, stable housing, healthy foods, and insurance coverage, which can lead to health disparities and higher risk for COVID-19 incidence among this aggregate population. We also suspect the notable rise in cluster 9 (since August) is due to multiple reasons including both dropping temperatures and the fact that it is located in the Midwestern US census region, which has been the epicenter of long-term care facility outbreaks during past four months from August to November 2020 according to [77].

**Fig 6:**
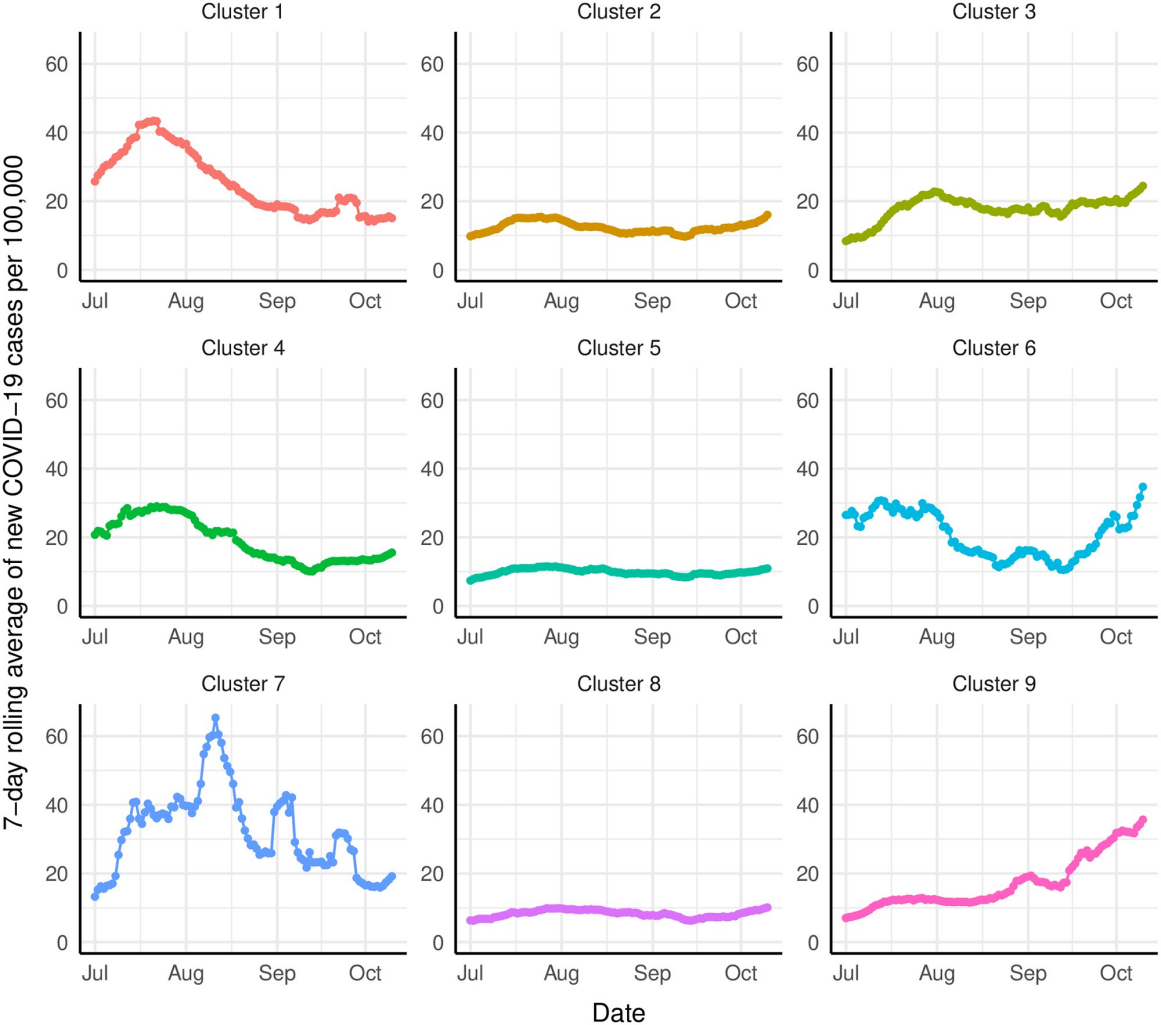
Cluster per capita COVID-19 case trends.

It is clear that the characteristics and trends are different for all of the defined clusters. Given the diversity from cluster to cluster, the underlying factors inherent to the associated groups affect both the speed and impact of the disease propagation. This inter-cluster diversity should be considered when designing interventions to effectively slow or stop the spread.

## Conclusion

Forecasting COVID-19 propagation is difficult. The challenge is exacerbated for projections focused on local regions and locations with smaller populations such as rural areas in the US. In part, this is due to the reliance of traditional methods on assumptions of population homogeneity. The heterogeneity of US counties contributes to this complexity and local factors may have disproportionate affect on disease spread.

The overall research objective of this study is to produce a new, statistically sound, data-driven clustering of US counties to create a novel COVID-19 related map of the US which balances issues of data quantity with that of regional diversity along a critical feature set. The resulting newly defined clusters are more homogeneous groups whose populations can be analyzed distinctly from one another. To achieve the objective, we address several important sub-tasks including (i) aggregation of a large array of demographic, mobility, health, and weather data, (ii) data transformation via dimension reduction to create a data set amenable to the research scope, and (iii) extensive experimentation with appropriate machine learning methods to intelligently filter and rank critical variables. From this exploration, we discover weather playing a dominant role in case propagation in a similar fashion as regular influenza spread; demonstrate that race plays an outsized role for both case counts and deaths; identify self-reported health and mental health as important predictors; find that there is some political bias that relates to recent increases in county-level cases. Finally (iv), using *k*-means, agglomerative hierarchical clustering, and Partitioning Around Medoids, we evaluate numerous county-level clustering outcomes to determine a final set with good mathematical properties (i.e., according to the Gap statistic) and that is composed of semi-contiguous regions that reflect wide diversity in their characteristics and COVID-19 patterns. Since this latter element was *not* embedded into the design of the clusters, the vastly different COVID-19 propagation trends are a direct result of the cluster definitions. This provides additional empirical evidence that the critical factors we identify do drive COVID-19 outcomes.

The policies, communication, and interventions to protect all groups identified should take into account their distinct profiles. This study provides a mechanism to leverage data to better understand the diversity across the nation and how that diversity impacts disease spread. When considering the clusters, meaningful patterns emerge that can help guide policy decisions, mitigation efforts, and analytical accuracy. In future work, we seek to leverage the unique characteristics of each cluster to enhance regional and local level time series forecasting and disease prediction. Additionally, we will consider the impact of local, state, and federal public health interventions on the unique subgroups across the US and how these exogenous factors interact with the inherent characteristics of the clusters to affect disease propagation.
